# Prenatal dexamethasone treatment for classic 21-hydroxylase deficiency in Europe

**DOI:** 10.1530/EJE-21-0554

**Published:** 2022-03-02

**Authors:** Hanna Nowotny, Uta Neumann, Véronique Tardy-Guidollet, S Faisal Ahmed, Federico Baronio, Tadej Battelino, Jérôme Bertherat, Oliver Blankenstein, Marco Bonomi, Claire Bouvattier, Aude Brac de la Perrière, Sara Brucker, Marco Cappa, Philippe Chanson, Hedi L Claahsen-van der Grinten, Annamaria Colao, Martine Cools, Justin H Davies, Helmut-Günther Dörr, Wiebke K Fenske, Ezio Ghigo, Roberta Giordano, Claus H Gravholt, Angela Huebner, Eystein Sverre Husebye, Rebecca Igbokwe, Anders Juul, Florian W Kiefer, Juliane Léger, Rita Menassa, Gesine Meyer, Vassos Neocleous, Leonidas A Phylactou, Julia Rohayem, Gianni Russo, Carla Scaroni, Philippe Touraine, Nicole Unger, Jarmila Vojtková, Diego Yeste, Svetlana Lajic, Nicole Reisch

**Affiliations:** 1 Medizinische Klinik und Poliklinik IV, Klinikum der Universität München, LMU München, Munich, Germany; 2 Centre for Chronic Sick Children, Department of Paediatric Endocrinology and Diabetology, Charité Universitätsmedizin Berlin, Berlin, Germany; 3 Laboratoire de Biochimie et Biologie Moléculaire, Hospices Civils de Lyon, Centre National de Référence ‘Développement Génital: du fœtus à l’adulte DEV-GEN’ Université Lyon I, Lyon, France; 4 Developmental Endocrinology Research Group, University of Glasgow, Glasgow, UK; 5 Paediatric Endocrinology Unit, Department of Medical and Surgical Sciences, S.Orsola-Malpighi University Hospital, Bologna, Italy; 6 Department of Endocrinology, Diabetes and Metabolic Diseases, University Medical Centre Ljubljana, University Children’s Hospital, Ljubljana, Slovenia; 7 Service d’Endocinologie et Maladies Métaboliques, Hôpitaux Universitaires Paris-Centre, Assistance Publique – Hôpitaux de Paris, Paris, France; 8 Department of Medical Biotechnology and Translational Medicine, University of Milan, Milan, Italy; 9 Department of Endocrine and Metabolic Diseases and Lab of Endocrine and Metabolic Research, IRCSS Istituto Auxologico Italiano, Milan, Italy; 10 Service d’Endocrinologie de l’Enfant, GHU Paris-Sud, Hôpital de Bicêtre, Paris, France; 11 Centre National de Référence ‘Développement Génital: du fœtus à l’adulte DEV-GEN’, Paris, France; 12 Fédération d’Endocrinologie, de Diabétologie et des Maladies Métaboliques, Hospices Civils des Lyon, Centre National de Référence ‘Développement Génital: du fœtus à l’adulte DEV-GEN’, Lyon, France; 13 Department of Women’s Health, University Women’s Hospital, University of Tübingen, Tübingen, Germany; 14 Endocrinology Unit, Paediatric University Department, Bambino Gesù Children’s Hospital, IRCCS, Rome, Italy; 15 Assistance Publique-Hôpitaux de Paris, Université Paris-Saclay, Service d’Endocrinologie et des Maladies de la Reproduction, Centre de Référence des Maladies Rares de Hypophyse, Hôpital Bicêtre, Le Kremlin-Bicêtre, France; 16 Department of Paediatric Endocrinology, Amalia Children’s Hospital, Radboud University Nijmegen Medical Centre, Nijmegen, The Netherlands; 17 Dipartimento Di Medicina Clinica E Chirurgia, Sezione Di Endocrinologia, Universita’ Federico II di Napoli, Naples, Italy; 18 Department of Paediatric Endocrinology, Ghent University Hospital, University of Ghent, Ghent, Belgium; 19 Paediatric Endocrinology, University Hospital Southampton NHS Foundation Trust, Southampton, UK; 20 Paediatric Endocrinology, Department of Paediatrics, Universitätsklinikum Erlangen, Erlangen, Germany; 21 Division of Endocrinology, Diabetes and Metabolism, Department of Internal Medicine I, University Hospital Bonn, Bonn, Germany; 22 Division of Endocrinology and Metabolism, Department of Internal Medicine, University of Turin, Turin, Italy; 23 Department of Endocrinology and Internal Medicine, Aarhus University Hospital, Aarhus, Denmark; 24 Klinik für Kinder- und Jugendmedizin, Universitätsklinikum Dresden, Technische Universität Dresden, Dresden, Germany; 25 Department of Clinical Science and KG Jebsen Centre for Autoimmune Disorders, University of Bergen, Bergen, Norway; 26 Department of Medicine, Haukeland University Hospital, Bergen, Norway; 27 West Midlands Regional Genetics Laboratory, Birmingham Women’s Hospital NHS Foundation Trust, Birmingham, UK; 28 Department of Growth and Reproduction, Copenhagen University Hospital – Rigshospitalet, Copenhagen, Denmark; 29 Department of Clinical Medicine, University of Copenhagen, Copenhagen, Denmark; 30 Clinical Division of Endocrinology and Metabolism, Department of Medicine III, Medical University of Vienna, Vienna, Austria; 31 Department of Paediatric Endocrinology and Diabetology and Reference Centre for Rare Diseases of Growth and Development, AP-HP Paris Nord Université de Paris, CHU Robert-Debre, Paris, France; 32 Division of Endocrinology, Department of Internal Medicine 1, Goethe University Frankfurt Faculty 16 Medicine, Frankfurt am Main, Germany; 33 Department of Molecular Genetics, Function and Therapy, The Cyprus Institute of Neurology and Genetics, Nicosia, Cyprus; 34 Cyprus School of Molecular Medicine, The Cyprus Institute of Neurology and Genetics, Nicosia, Cyprus; 35 Centre of Reproductive Medicine and Andrology, Clinical and Operative Andrology, University of Münster, Münster, Germany; 36 Department of Paediatrics, Endocrine Unit, Scientific Institute San Raffaele, Milan, Italy; 37 Dipartimento di Medicina, U.O.C. Endocrinologia, Università di Padova, Padova, Italy; 38 Department of Endocrinology and Reproductive Medicine, Centre for Rare Endocrine and Gynaecological Disorders, Sorbonne Université, Assistance Publique Hopitaux de Paris, Paris, France; 39 Department of Endocrinology, Diabetes and Metabolism, University Hospital Essen, Essen, Germany; 40 Department of Paediatrics, Jessenius Faculty of Medicine, Comenius University in Bratislava, University Hospital in Martin, Martin, Slovakia; 41 Paediatric Endocrinology Service, Hospital Universitari Vall d’Hebron, Barcelona, Spain; 42 Autonomous University of Barcelona, Bellaterra, Spain; 43 CIBERER, ISCIII, Madrid, Spain; 44 Department of Women’s and Children’s Health, Karolinska Institutet/Karolinska University Hospital, Paediatric Endocrinology Unit (QB83), Stockholm, Sweden

## Abstract

**Objective:**

To assess the current medical practice in Europe regarding prenatal dexamethasone (Pdex) treatment of congenital adrenal hyperplasia (CAH) due to 21-hydroxylase deficiency.

**Design and methods:**

A questionnaire was designed and distributed, including 17 questions collecting quantitative and qualitative data. Thirty-six medical centres from 14 European countries responded and 30 out of 36 centres were reference centres of the European Reference Network on Rare Endocrine Conditions, EndoERN.

**Results:**

Pdex treatment is currently provided by 36% of the surveyed centres. The treatment is initiated by different specialties, that is paediatricians, endocrinologists, gynaecologists or geneticists. Regarding the starting point of Pdex, 23% stated to initiate therapy at 4–5 weeks postconception (wpc), 31% at 6 wpc and 46 % as early as pregnancy is confirmed and before 7 wpc at the latest. A dose of 20 µg/kg/day is used. Dose distribution among the centres varies from once to thrice daily. Prenatal diagnostics for treated cases are conducted in 72% of the responding centres. Cases treated per country and year vary between 0.5 and 8.25. Registries for long-term follow-up are only available at 46% of the centres that are using Pdex treatment. National registries are only available in Sweden and France.

**Conclusions:**

This study reveals a high international variability and discrepancy in the use of Pdex treatment across Europe. It highlights the importance of a European cooperation initiative for a joint international prospective trial to establish evidence-based guidelines on prenatal diagnostics, treatment and follow-up of pregnancies at risk for CAH.

## Introduction

Androgen excess in girls with congenital adrenal hyperplasia (CAH) results in virilization of the external genitalia of varying degree (1, 2, 3, 4, 5, 6). Surgeries, such as correction of the urogenital sinus (7, 8, 9), may lead to psychological and psychosexual issues in adult life, such as impaired genital sensitivity, sexual dysfunction and urinary incontinence (10, 11, 12, 13, 14). Dexamethasone (15) at a dose of 20 µg/kg/day initiated before 6–7 weeks postconception (wpc), that is the critical window of sexual differentiation, traverses the placenta, is able to suppress fetal androgen production and hence has been shown to effectively prevent or reduce prenatal virilization (6, 16, 17, 18, 19). It has been in use since 1984 but its use is highly debated for several reasons (20).

First, it holds an ethical dilemma since unaffected fetuses currently are treated unnecessarily during the first trimester of fetal life. Genetic diagnosis can in most countries only be established at 1012 weeks wpc by chorionic villous sampling (21, 22, 23). This translates to a risk of only one in eight fetuses benefitting from prenatal dex treatment (Pdex) or at least one in four if non-invasive sex determination is performed (18).

Secondly, and most importantly, there is not enough evidence for the safety of treated fetuses. Aberrant fetal programming with effects on the cardiovascular system, metabolism and cognitive performance have been described in both animal studies and in humans (24, 25, 26). The clinical outcome studies of Pdex show conflicting results. Some studies show no negative effect on neuropsychological functions and behaviour in non-CAH patients who have been exposed to Pdex treatment in the first trimester of fetal life (27, 28, 29, 30). A survey based on parental questionnaires of prenatally Pdex-exposed children with and without CAH compared to unexposed children did not indicate any adverse effects regarding motor and cognitive development (31). Other studies show that early Pdex treatment in individuals without CAH affects cognition and behaviour during childhood (32, 33, 34, 35, 36, 37) as well as the methylation pattern of the genome (38) and insulin secretion (26) and that the effects are stronger in girls (26, 33). Altered brain structures (39) and insulin secretion (25, 26) have also been identified during adulthood. In a small cohort of women with CAH treated with dex during the entire gestational period a negative effect on cognition was observed compared to an untreated female CAH cohort (40). During childhood, lower intellectual ability was observed in girls with CAH when treated with Pdex (36, 41). Another study showed improved cognitive development in CAH-affected girls treated with Pdex, however, unfavorable cognitive functions in female CAH-unaffected patients were identified in the same report (37).

Thirdly, only few data on the mothers’ safety are available so far. The available literature has highlighted the adverse effects of glucocorticoid excess (16, 21, 42, 43). Strong opinions on this controversial experimental treatment (44, 45) have been raised, yet only a few countries have evaluated the effect and plausible side effects of Pdex in a systematic way in their own population, but still, centres in Europe do use Pdex. According to the Endocrine Society its use is to be restricted to institutional review board-approved research settings (5, 46). In Sweden, the practice of Pdex has been put on hold due to the negative findings in non-CAH first trimester treated cases (47).

The aim of this study is to offer a current reflection of the international views on and practice of Pdex in CAH across Europe and to highlight the importance of longitudinal follow-up of treated cases and prospective clinical trials that investigate different aspects of this therapy.

## Methods

This study/survey did not involve patients, thus no patient consent was necessary. The study was approved by the ethics committee of the Medical Faculty of the Ludwig-Maximilians-Universität München, Munich, Germany (project no 21-0760).

### Questionnaire design

A questionnaire was designed and distributed using Microsoft Forms (Microsoft Office 365, Windows 10, Microsoft). Seventeen questions were designed including a mixture of open questions and dichotomous or multiple-choice questions with either a single or multiple answer possibilities.

### Study group

A total of 45 centres were contacted for completion of the questionnaire. These comprised all European Reference Network on Rare Endocrine Conditions (EndoERN) healthcare centres part of the main thematic groups ‘adrenal’ and/or ‘sex development and maturation’. Additionally, we included a few (*n* = 6) further tertiary care centres with specific expertise in the field but not yet certified as an EndoERN reference centre. The questionnaire was completed by 80% (36/45) of these endocrine tertiary care centres across Europe, of which 83% (30/36) are reference centres of EndoERN and all are certified centres for adrenal conditions.

### Data extraction and analysis

Data extraction was performed using Microsoft Excel. Prism version 8 (GraphPad Software) and Adobe Illustrator 24.3 2020 (Adobe) were used for statistical analysis and graphical presentation of the results.

## Results

### Characteristics of study cohort

**Figure 1:**
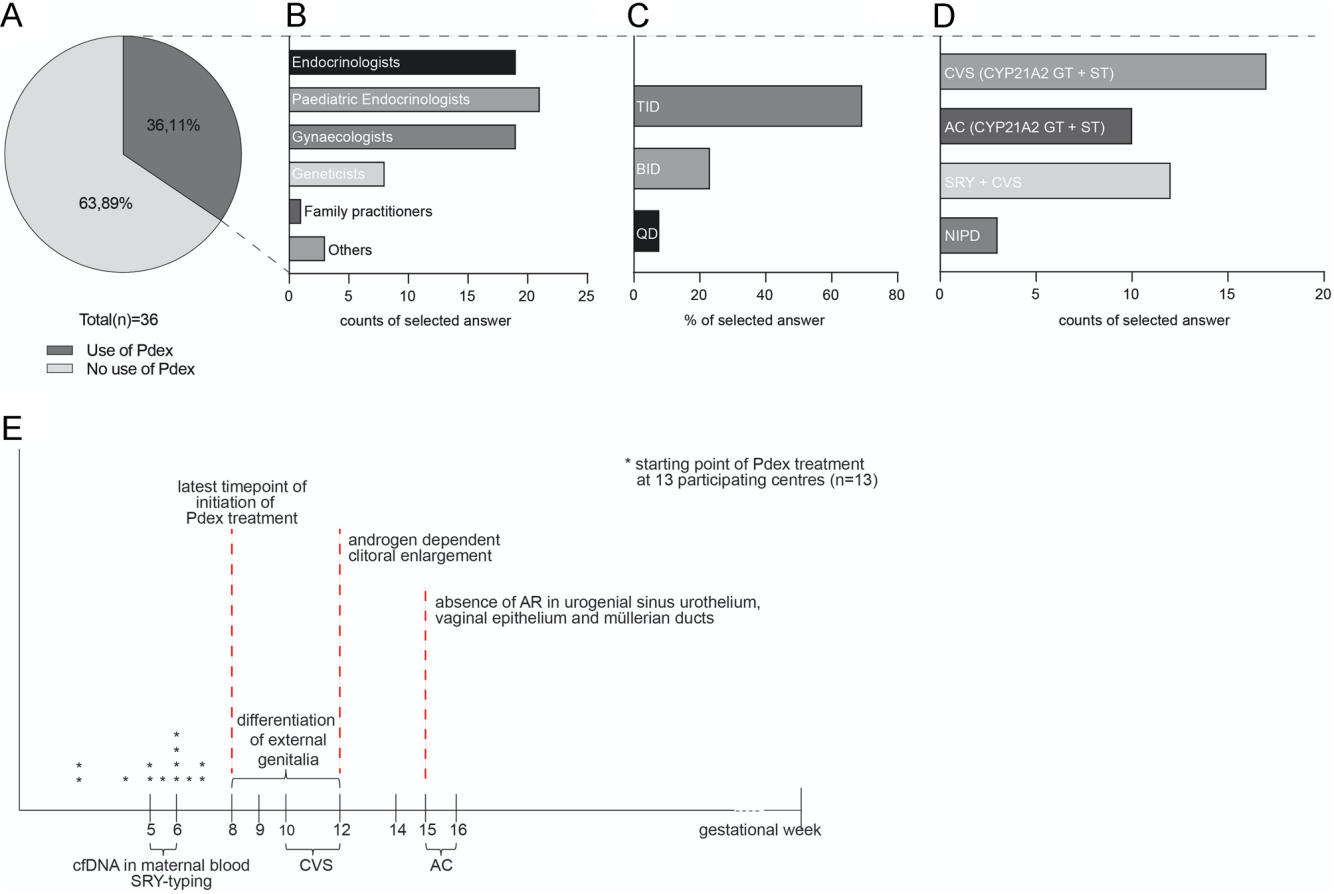
Use of Pdex treatment and prenatal diagnostics to prevent virilization in girls with CAH. (A) Pie chart depicting the percentage of included centres using or not using Pdex treatment (*n* = 36). (B) Selected disciplines providing Pdex treatment in the corresponding country. Multiple selections were possible (*n* = 31; 5 NA). (C) Daily dosing distribution of Pdex. Only centres using Pdex treatment were included (*n* = 13). (D) Types of prenatal diagnostics for CAH used in each corresponding country (*n* = 25). CVS (*CYP21A2* GT + ST): *CYP21A2* genotyping and sextyping between 1012 wpc; treatment is discontinued for male foetuses or notaffected females. AC (*CYP21A2* GT + ST): *CYP21A2* genotyping and sextyping between 1516 wpc; treatment is discontinued for male foetuses or notaffected females. *SRY* + CVS: *SRY*typing from maternal blood (cfDNA); treatment of females only; CVS for *CYP21A2* GT between 1012 wpc; treatment only continued for affected females. NIPD: massively parallel sequencing using cfDNA from maternal blood; only affected females are treated. (E) Overview of important timepoints of development of external genitalia in females. Stars are marking the starting point of Pdex treatment as indicated by each centre (*n* = 13).

The questionnaire entitled ‘Prenatal dexamethasone treatment in CAH across Europe’ was completed by a total of 36 medical centres across 14 different European countries (Table 1). Currently, 36% (13/36) of the listed medical centres provide Pdex (Fig. 1). As depicted in Table 1, Pdex is applied in 0.5–8.25 cases per country and year. This data mostly depended on individual estimation and is only in the minority of cases generated by hospital or disease registries. The median number of pregnant women who received Pdex during the first trimester of pregnancy per centre was ten (*n* = 197) and a lower median amount of five women per centre received Pdex for the entire gestational period (*n* = 72).

**Table 1:** Countries and centres included in the questionnaire ‘Prenatal dexamethasone treatment in CAH across Europe’ and numbers of treated pregnancies per year and per total time since initiation of treatment.

Country (*n* = 14)	Centers/Country (*n* = 36)	Use of pdex (N/N centres)	Number of PDEX cases/year		Total number of pregnancies	
			Estimated	Reported	First trimester	Entire pregnancy
Austria	1	0				
Belgium	1	0				
Cyprus	1	0				
Denmark	2	0				
France	5	3	4	2	64	21
Germany	10	5	8.25		38	27
Italy	7	2	3.5		60	10
Netherlands	1	1	1		10	5
Norway	1	0				
Slovakia	1	0				
Slovenia	1	0				
Spain	1	1		0.5	18	3
Sweden	1	0				
UK	3	1	2		7	6

### Current use of Pdex across Europe

The majority of countries listed Pdex being provided not by a single, but different specialties (paediatric endocrinologists, endocrinologists and gynaecologists/obstetricians and in rare cases also by geneticists; Fig. 1B). Of all centres using Pdex, there was 100% congruency on the recommended dose of 20 µg/kg/day; however, the daily dose distribution varied. Most medical centres (9/13, 69%) use thrice daily (TID) application of dex. Twice daily (BID) application is used by 23% (3/13) of centres and once daily (QD) dex application is used by one of the surveyed medical centres (Fig. 1C). Regarding the starting point of Pdex responses showed at least some congruency with 23% of centres initiating therapy at 4 to 5 wpc, 31% at 6 wpc, and 46% as early as pregnancy is confirmed and before 7 wpc at the latest (Fig. 1E). Prenatal diagnostics for CAH in treated cases are conducted at 72% (26/36) of recruited centres. For the question regarding the types of prenatal diagnostics used at each centre, multiple answers were possible. The majority (65 % of centres, 41 % of answers) uses chorionic villus sampling (CVS) including *CYP21A2* genotyping (*CYP21A2* GT) and sex typing between the gestational week (GW) 1012, whereas 38% of centres (24% of answers) uses amniocentesis (AC) including *CYP21A2* GT and sex typing in GW 1516. Genotyping of the sex-determining region Y (*SRY*typing) from maternal blood (GW 5–7) combined with CVS+*CYP21A2* GT at GW 10–12 is used by 46% of centres (29% of answers). Early non-invasive prenatal diagnostics (NIPD) using the combination of *SRY*typing and *CYP21A2* GT by massively parallel sequencing of cell-free fetal DNA in maternal blood (cfDNA) is offered by only one of the surveyed centres (Birmingham, UK). Of the 13 centres providing Pdex treatment, 11/13 centres offer *SRY*typing+CVS and *CYP21A2* GT (Fig. 1D).

## Discussion

This study offers a current cross-sectional status quo of Pdex in CAH in different tertiary care centres across Europe.

The data obtained demonstrate that only approximately a third of centres included in the study are actually providing Pdex (Fig. 1A). Follow-up on treated Pdex cases for both 21OHD and treated unaffected children with a rate of only 42% (15/36) and availability of registries for prenatally treated cases at only 46% (6/13) of the centres applying experimental Pdex is unacceptable. Moreover, only a small number of cases are treated at each centre per year with a minimum of approximately one case per every 4 years to three cases per year (Table 1). National registries were reported to be available in Sweden, Italy, France and Germany, however, only in Sweden and France they are population based. The total number of treated cases in other European countries can only be estimated. The Swedish PREDEX database registered 276 treated cases and untreated controls in Sweden and Italy within 10 years. In Germany, in a period of 10 years, 148 Pdex treated cases have been documented based on voluntary reporting of the treating physicians; however, the data has not been published due to incomplete documentation. In France, over a period of 9 years (2002–2011), a total of 258 fetuses at risk of CAH were subjected to early non-invasive sex determination (18). After the exclusion of male fetuses, 154 of them were subjected to Pdex. Currently, in France, a multicentric study is investigating somatic, neurocognitive and metabolic outcomes of Pdex-treated subjects. Based on these data at least 10 prenatal treatments per year can be expected in Germany and France. This highlights the strong need and additional benefit of a European collaborative initiative, which was expressed by 75% (27/36) of research centres.

Our data show that a substantial number of cases are treated outside the adrenal endocrine tertiary care centres that were approached in this questionnaire and that treatment is initiated by various disciplines. In such an ultra-rare and complex condition, this is alarming. We propose to channel this experimental treatment by international study protocols to endocrine expert centres with adequate long-term follow-up in order to disclose potential side effects.

The critical time point of differentiation of external genitalia is between 8 and 12 wpc or even from 6 wpc onwards (6, 7, 48). To safeguard the development of female genitalia and prevent virilization, dex administration is required as early as from 6 wpc onwards up to at least 16 wpc (49). Analysis of current practice in Europe indicates that centres conducting Pdex usually start treatment as early as pregnancy is confirmed or before 6/7 wpc at the latest (Fig. 1E). Of the estimated 269 cases treated at all included centres since starting point of Pdex at each individual centre, most cases were only treated for the time period of the first trimester of pregnancy (*n*  = 197; 73 %), whereas 72 cases (27 %) received full-term treatment (Table 1). Currently, a dose of 20 µg/kg/day is used by all centres surveyed, however, distribution regarding multiple doses during the day varies (Fig. 1C). In 2006, the idea was promoted of reducing dex dosage after 16 wpc in order to decrease adverse maternal effects of glucocorticoid therapy without compromising treatment efficacy (49). A recent publication even stated the traditional Pdex dose being three-fold higher than actually needed and suggested TID administration to allow for more stable plasma concentrations (50).

Another important aspect refers to prenatal diagnostics in fetuses potentially affected with classic CAH. Despite the improvement of CVS and AC, which is used in most cases, there is still a small risk of miscarriage in the first 23 weeks of pregnancy due to diagnostic-related complications (51). A combinational approach of early NIPD *SRY*typing from maternal plasma (GW5-6) and CVS in GW10-12 is offered by 11/13 centres providing Pdex and therefore preventing boys from unnecessary prenatal treatment. In France, boys have not been treated anymore for several years. Thus SRY-typing is recommended to reduce the number of treated fetuses but does not solve the entire problem. Targeted massively parallel sequencing of cell-free DNA from plasma drawn from an expectant mother (52, 53) and *SRY*typing (18) to determine sex and *CYP21A2* GT as early as 6 wpc; however, is only provided by one of the included centres. This strategy could efficiently prevent needless treatment of unaffected children and discard the risk of miscarriage.

## Conclusion

Current medical standards regarding Pdex are lacking evidence-based guidelines on the optimal starting point, optimal duration and optimal dosing as well as standardized surveillance and follow-up at specialized centres. An international collaborative initiative on a prospective randomized trial is needed to allow for sufficient sample sizes in order to answer the key questions of this therapy to allow its future use or to ban it.

## Declaration of interest

The authors declare that there is no conflict of interest that could be perceived as prejudicing the impartiality of this article.

## Funding

This work was supported by the Deutsche Forschungsgemeinschaft (Heisenberg Professorship, 325768017 to N R and 314061271-TRR205 to N R and A H), the European Commission for funding EndoERN CHAFEA FPA grant no. 739527, the Eva Luise und Horst Köhler Stiftung & Else Kröner-Fresenius-Stiftung (2019_KollegSE.03 to H N) and the Stockholm County Council (Senior clinical research fellowship dnr RS 2019-1140 to S L), Stiftelsen Frimurare Barnhuset i Stockholm and Lisa and Johan Grönbergs Stiftelse.

## Author contribution statement

S Lajic and N Reisch contributed equally to this work.

## References

[1] Therrell BL Newborn screening for congenital adrenal hyperplasia. Endocrinology and Metabolism Clinics of North America 2001 30 15–30. (10.1016/s0889-8529(0870017-3) 11344933

[2] Reisch N Willige M Kohn D Schwarz HP Allolio B Reincke M Quinkler M Hahner S Beuschlein F. Frequency and causes of adrenal crises over lifetime in patients with 21-hydroxylase deficiency. European Journal of Endocrinology 2012 167 35–42. (10.1530/EJE-12-0161) 22513882

[3] Zetterstrom RH Karlsson L Falhammar H Lajic S Nordenstrom A. Update on the Swedish newborn screening for congenital adrenal hyperplasia due to 21-hydroxylase deficiency. International Journal of Neonatal Screening 2020 6 71. (10.3390/ijns6030071) PMC7570065 33239597

[4] Speiser PW Congenital adrenal hyperplasia owing to 21-hydroxylase deficiency. Endocrinology and Metabolism Clinics of North America 2001 30 31–59, vi. (10.1016/s0889-8529(0870018-5) 11344938

[5] Speiser PW Azziz R Baskin LS Ghizzoni L Hensle TW Merke DP Meyer-Bahlburg HF Miller WL Montori VM Oberfield SE Congenital adrenal hyperplasia due to steroid 21-hydroxylase deficiency: an Endocrine Society clinical practice guideline. Journal of Clinical Endocrinology and Metabolism 2010 95 4133–4160. (10.1210/jc.2009-2631) 20823466 PMC2936060

[6] Goto M Piper Hanley K Marcos J Wood PJ Wright S Postle AD Cameron IT Mason JI Wilson DI Hanley NA. In humans, early cortisol biosynthesis provides a mechanism to safeguard female sexual development. Journal of Clinical Investigation 2006 116 953–960. (10.1172/JCI25091) PMC1421344 16585961

[7] Hanley NA Arlt W. The human fetal adrenal cortex and the window of sexual differentiation. Trends in Endocrinology and Metabolism 2006 17 391–397. (10.1016/j.tem.2006.10.001) 17046275

[8] Binet A Lardy H Geslin D Francois-Fiquet C Poli-Merol ML. Should we question early feminizing genitoplasty for patients with congenital adrenal hyperplasia and XX karyotype? Journal of Pediatric Surgery 2016 51 465–468. (10.1016/j.jpedsurg.2015.10.004) 26607969

[9] Elsayed S Badawy H Khater D Abdelfattah M Omar M. Congenital adrenal hyperplasia: does repair after two years of age have a worse outcome? Journal of Pediatric Urology 2020 16 424.e1–424.e6. (10.1016/j.jpurol.2020.06.010) 32712187

[10] Simpson JL Rechitsky S. Prenatal genetic testing and treatment for congenital adrenal hyperplasia. Fertility and Sterility 2019 111 21–23. (10.1016/j.fertnstert.2018.11.041) 30611408

[11] Meyer-Bahlburg HFL Khuri J Reyes-Portillo J Ehrhardt AA New MI. Stigma associated with classical congenital adrenal hyperplasia in women’s sexual lives. Archives of Sexual Behavior 2018 47 943–951. (10.1007/s10508-017-1003-8) 28523454

[12] Crouch NS Minto CL Laio LM Woodhouse CR Creighton SM. Genital sensation after feminizing genitoplasty for congenital adrenal hyperplasia: a pilot study. BJU International 2004 93 135–138. (10.1111/j.1464-410x.2004.04572.x) 14678385

[13] Gastaud F Bouvattier C Duranteau L Brauner R Thibaud E Kutten F Bougneres P. Impaired sexual and reproductive outcomes in women with classical forms of congenital adrenal hyperplasia. Journal of Clinical Endocrinology and Metabolism 2007 92 1391–1396. (10.1210/jc.2006-1757) 17284631

[14] Nordenstrom A Frisen L Falhammar H Filipsson H Holmdahl G Janson PO Thoren M Hagenfeldt K Nordenskjold A. Sexual function and surgical outcome in women with congenital adrenal hyperplasia due to CYP21A2 deficiency: clinical perspective and the patients’ perception. Journal of Clinical Endocrinology and Metabolism 2010 95 3633–3640. (10.1210/jc.2009-2639) 20466782

[15] Dexter PM Caldwell KA Caldwell GA. A predictable worm: application of Caenorhabditis elegans for mechanistic investigation of movement disorders. Neurotherapeutics 2012 9 393–404. (10.1007/s13311-012-0109-x) 22403010 PMC3337026

[16] Forest MG David M Morel Y. Prenatal diagnosis and treatment of 21-hydroxylase deficiency. Journal of Steroid Biochemistry and Molecular Biology 1993 45 75–82. (10.1016/0960-0760(9390125-g) 8481354

[17] Forest MG Betuel H David M. Prenatal treatment in congenital adrenal hyperplasia due to 21-hydroxylase deficiency: up-date 88 of the French multicentric study. Endocrine Research 1989 15 277–301. (10.1080/07435808909039101) 2667968

[18] Tardy-Guidollet V Menassa R Costa JM David M Bouvattier-Morel C Baumann C Houang M Lorenzini F Philip N Odent S New management strategy of pregnancies at risk of congenital adrenal hyperplasia using fetal sex determination in maternal serum: French cohort of 258 cases (2002–2011). Journal of Clinical Endocrinology and Metabolism 2014 99 1180–1188. (10.1210/jc.2013-2895) 24471566

[19] Gorduza D Tardy-Guidollet V Robert E Gay CL Chatelain P David M Bretones P Lienhardt-Roussie A Brac de la Perriere A Morel Y Late prenatal dexamethasone and phenotype variations in 46,XX CAH: concerns about current protocols and benefits for surgical procedures. Journal of Pediatric Urology 2014 10 941–947. (10.1016/j.jpurol.2014.02.003) 24679821

[20] David M Forest MG. Prenatal treatment of congenital adrenal hyperplasia resulting from 21-hydroxylase deficiency. Journal of Pediatrics 1984 105 799–803. (10.1016/s0022-3476(8480310-8) 6334149

[21] New MI Carlson A Obeid J Marshall I Cabrera MS Goseco A Lin-Su K Putnam AS Wei JQ Wilson RC. Prenatal diagnosis for congenital adrenal hyperplasia in 532 pregnancies. Journal of Clinical Endocrinology and Metabolism 2001 86 5651–5657. (10.1210/jcem.86.12.8072) 11739415

[22] Forest MG Recent advances in the diagnosis and management of congenital adrenal hyperplasia due to 21-hydroxylase deficiency. Human Reproduction Update 2004 10 469–485. (10.1093/humupd/dmh047) 15514016

[23] Mercado AB Wilson RC Cheng KC Wei JQ New MI. Prenatal treatment and diagnosis of congenital adrenal hyperplasia owing to steroid 21-hydroxylase deficiency. Journal of Clinical Endocrinology and Metabolism 1995 80 2014–2020. (10.1210/jcem.80.7.7608248) 7608248

[24] Khulan B Drake AJ. Glucocorticoids as mediators of developmental programming effects. Best Practice and Research: Clinical Endocrinology and Metabolism 2012 26 689–700. (10.1016/j.beem.2012.03.007) 22980050

[25] Riveline JP Baz B Nguewa JL Vidal-Trecan T Ibrahim F Boudou P Vicaut E Brac de la Perriere A Fetita S Breant B Exposure to glucocorticoids in the first part of fetal life is associated with insulin secretory defect in adult humans. Journal of Clinical Endocrinology and Metabolism 2020 105 dgz145. (10.1210/clinem/dgz145) 31665349

[26] Wallensteen L Karlsson L Messina V Nordenstrom A Lajic S. Perturbed beta-cell function and lipid profile after early prenatal dexamethasone exposure in individuals without CAH. Journal of Clinical Endocrinology and Metabolism 2020 105 e2439–e2448. (10.1210/clinem/dgaa280) PMC7343997 32433752

[27] Van’t Westeinde A Zimmermann M Messina V Karlsson L Padilla N Lajic S. First trimester DEX treatment is not associated with altered brain activity during working memory performance in adults. Journal of Clinical Endocrinology and Metabolism 2020 105 e4074–e4082. 10.1210/clinem/dgaa611 PMC7510958 32869847

[28] Karlsson L Nordenstrom A Hirvikoski T Lajic S. Prenatal dexamethasone treatment in the context of at risk CAH pregnancies: long-term behavioral and cognitive outcome. Psychoneuroendocrinology 2018 91 68–74. (10.1016/j.psyneuen.2018.02.033) 29529521

[29] Wallensteen L Karlsson L Messina V Gezelius A Sandberg MT Nordenstrom A Hirvikoski T Lajic S. Evaluation of behavioral problems after prenatal dexamethasone treatment in Swedish children and adolescents at risk of congenital adrenal hyperplasia. Hormones and Behavior 2018 98 219–224. (10.1016/j.yhbeh.2017.11.004) 29410007

[30] Hirvikoski T Nordenstrom A Lindholm T Lindblad F Ritzen EM Lajic S. Long-term follow-up of prenatally treated children at risk for congenital adrenal hyperplasia: does dexamethasone cause behavioural problems? European Journal of Endocrinology 2008 159 309–316. (10.1530/EJE-08-0280) 18579553

[31] Meyer-Bahlburg HF Dolezal C Baker SW Carlson AD Obeid JS New MI. Cognitive and motor development of children with and without congenital adrenal hyperplasia after early-prenatal dexamethasone. Journal of Clinical Endocrinology and Metabolism 2004 89 610–614. (10.1210/jc.2002-021129) 14764770

[32] Hirvikoski T Lindholm T Lajic S Nordenstrom A. Gender role behaviour in prenatally dexamethasone-treated children at risk for congenital adrenal hyperplasia – a pilot study. Acta Paediatrica 2011 100 e112–e119. (10.1111/j.1651-2227.2011.02260.x) 21388450

[33] Wallensteen L Zimmermann M Thomsen Sandberg M Gezelius A Nordenstrom A Hirvikoski T Lajic S. Sex-dimorphic effects of prenatal treatment with dexamethasone. Journal of Clinical Endocrinology and Metabolism 2016 101 3838–3846. (10.1210/jc.2016-1543) 27482827

[34] Hirvikoski T Nordenstrom A Lindholm T Lindblad F Ritzen EM Wedell A Lajic S. Cognitive functions in children at risk for congenital adrenal hyperplasia treated prenatally with dexamethasone. Journal of Clinical Endocrinology and Metabolism 2007 92 542–548. (10.1210/jc.2006-1340) 17148562

[35] Trautman PD Meyer-Bahlburg HF Postelnek J New MI. Effects of early prenatal dexamethasone on the cognitive and behavioral development of young children: results of a pilot study. Psychoneuroendocrinology 1995 20 439–449. (10.1016/0306-4530(9400070-0) 8532827

[36] Meyer-Bahlburg HF Dolezal C Haggerty R Silverman M New MI. Cognitive outcome of offspring from dexamethasone-treated pregnancies at risk for congenital adrenal hyperplasia due to 21-hydroxylase deficiency. European Journal of Endocrinology 2012 167 103–110. (10.1530/EJE-11-0789) 22549088 PMC3383400

[37] Maryniak A Ginalska-Malinowska M Bielawska A Ondruch A. Cognitive and social function in girls with congenital adrenal hyperplasia – influence of prenatally administered dexamethasone. Child Neuropsychology 2014 20 60–70. (10.1080/09297049.2012.745495) 23186079

[38] Karlsson L Barbaro M Ewing E Gomez-Cabrero D Lajic S. Epigenetic alterations associated with early prenatal dexamethasone treatment. Journal of the Endocrine Society 2019 3 250–263. (10.1210/js.2018-00377) 30623163 PMC6320242

[39] Van’t Westeinde A Karlsson L Nordenstrom A Padilla N Lajic S. First-trimester prenatal dexamethasone treatment is associated with alterations in brain structure at adult age. Journal of Clinical Endocrinology and Metabolism 2020 105 dgaa340. (10.1210/clinem/dgaa340) PMC7304558 32497228

[40] Karlsson L Gezelius A Nordenstrom A Hirvikoski T Lajic S. Cognitive impairment in adolescents and adults with congenital adrenal hyperplasia. Clinical Endocrinology 2017 87 651–659. (10.1111/cen.13441) 28771762

[41] Messina V Karlsson L Hirvikoski T Nordenstrom A Lajic S. Cognitive function of children and adolescents with congenital adrenal hyperplasia: importance of early diagnosis. Journal of Clinical Endocrinology and Metabolism 2020 105 e683–e691. (10.1210/clinem/dgaa016) PMC7343998 31927590

[42] Pang S Clark AT Freeman LC Dolan LM Immken L Mueller OT Stiff D Shulman DI. Maternal side effects of prenatal dexamethasone therapy for fetal congenital adrenal hyperplasia. Journal of Clinical Endocrinology and Metabolism 1992 75 249–253. (10.1210/jcem.75.1.1619017) 1619017

[43] Lajic S Wedell A Bui TH Ritzen EM Holst M. Long-term somatic follow-up of prenatally treated children with congenital adrenal hyperplasia. Journal of Clinical Endocrinology and Metabolism 1998 83 3872–3880. (10.1210/jcem.83.11.5233) 9814461

[44] Miller WL Fetal endocrine therapy for congenital adrenal hyperplasia should not be done. Best Practice and Research: Clinical Endocrinology and Metabolism 2015 29 469–483. (10.1016/j.beem.2015.01.005) 26051303

[45] Lajic S Nordenstrom A Hirvikoski T. Long-term outcome of prenatal dexamethasone treatment of 21-hydroxylase deficiency. Endocrine Development 2011 20 96–105. (10.1159/000321228) 21164263

[46] Clayton PE Miller WL Oberfield SE Ritzen EM Sippell WG Speiser PW & ESPE/LWPES CAH Working Group. Consensus statement on 21-hydroxylase deficiency from the European Society for Paediatric Endocrinology and the Lawson Wilkins Pediatric Endocrine Society. Hormone Research 2002 58 188–195. (10.1159/000065490) 12324718

[47] Hirvikoski T Nordenstrom A Wedell A Ritzen M Lajic S. Prenatal dexamethasone treatment of children at risk for congenital adrenal hyperplasia: the Swedish experience and standpoint. Journal of Clinical Endocrinology and Metabolism 2012 97 1881–1883. (10.1210/jc.2012-1222) 22466333

[48] Shapiro E Huang HY Wu XR. Uroplakin and androgen receptor expression in the human fetal genital tract: insights into the development of the vagina. Journal of Urology 2000 164 1048–1051. (10.1097/00005392-200009020-00031) 10958738

[49] White PC Ontogeny of adrenal steroid biosynthesis: why girls will be girls. Journal of Clinical Investigation 2006 116 872–874. (10.1172/JCI28296) PMC1421368 16585958

[50] Stachanow V Neumann U Blankenstein O Fuhr U Huisinga W Michelet R Reisch N Kloft C. Rationale of a lower dexamethasone dose in prenatal congenital adrenal hyperplasia therapy based on pharmacokinetic modelling. European Journal of Endocrinology 2021 185 365–374. (10.1530/EJE-21-0395) 34228630

[51] Alfirevic Z Navaratnam K Mujezinovic F. Amniocentesis and chorionic villus sampling for prenatal diagnosis. Cochrane Database of Systematic Reviews 2017 9 CD003252. (10.1002/14651858.CD003252.pub2) PMC6483702 28869276

[52] New MI Tong YK Yuen T Jiang P Pina C Chan KC Khattab A Liao GJ Yau M Kim SM Noninvasive prenatal diagnosis of congenital adrenal hyperplasia using cell-free fetal DNA in maternal plasma. Journal of Clinical Endocrinology and Metabolism 2014 99 E1022–E1030. (10.1210/jc.2014-1118) 24606108 PMC4037720

[53] Zhang J Li J Saucier JB Feng Y Jiang Y Sinson J McCombs AK Schmitt ES Peacock S Chen S Non-invasive prenatal sequencing for multiple Mendelian monogenic disorders using circulating cell-free fetal DNA. Nature Medicine 2019 25 439–447. (10.1038/s41591-018-0334-x) 30692697

